# Area-Level Social Deprivation and Cytomegalovirus Seropositivity at the Time of Solid Organ Transplant

**DOI:** 10.1001/jamanetworkopen.2024.37878

**Published:** 2024-10-07

**Authors:** Maheen Z. Abidi, Rocio Lopez, Susana Arrigain, Adriana Weinberg, Bruce Kaplan, Mara McAdams-DeMarco, Jesse D. Schold, Kristine M. Erlandson

**Affiliations:** 1 Division of Infectious Diseases, Department of Medicine, University of Colorado Denver School of Medicine; 2 Department of Surgery, University of Colorado Denver School of Medicine; 3 Colorado Center for Transplantation Care, Research and Education, Aurora; 4 Division of Pediatric Infectious Diseases, Departments of Pediatrics, Medicine, and Pathology, University of Colorado Denver School of Medicine; 5 Department of Surgery, NYU Grossman School of Medicine and Langone Health, New York, New York; 6 Department of Population Health, NYU Grossman School of Medicine, New York, New York

## Abstract

**Importance:**

Cytomegalovirus (CMV) is associated with significant morbidity and mortality in solid organ transplant (SOT) recipients. The risk factors for CMV seropositivity in SOT recipients, including area-level social deprivation in the US, have not been fully characterized.

**Objective:**

To (1) evaluate CMV seroprevalence, (2) assess the recipient characteristics associated with CMV seropositivity, and (3) assess the association of area-level social deprivation index (SDI) scores with pretransplant CMV serostatus.

**Design, Setting, and Participants:**

This retrospective cross-sectional analysis of the Scientific Registry of Transplant Recipients database included all adult (aged ≥18 years) SOT recipients from January 1, 2008, to May 31, 2022. Data were analyzed from April 10 to October 25, 2023.

**Exposure:**

Recipient characteristics and area-level SDI.

**Main Outcomes and Measures:**

Multivariable generalized linear models were used to evaluate the association between (1) patient characteristics and CMV and (2) social deprivation (measured by SDI scores, which were assessed in quintiles, from lowest to highest) and CMV seropositivity. In addition, differences based on patient demographics and the transplanted organ(s) were evaluated.

**Results:**

Among the 389 288 SOT recipients included in the analysis, mean (SD) age was 53.3 (13.0) years; 63.0% were male, 21.4% were Black, 15.2% were Hispanic White, 56.2% were non-Hispanic White, and 62.7% were CMV seropositive. The mean (SD) age was higher among CMV seropositive (54.0 [12.7] years) compared with CMV seronegative (52.0 [13.5] years) patients. Seropositivity for CMV was higher among women (69.9%) than men (58.5%) and among Black (74.8%) and Hispanic White (80.2%) patients compared with non-Hispanic White patients (50.4%). Seropositivity for CMV was highest among kidney (64.5%), liver (63.6%), and kidney and liver (66.2%) recipients. Greater SDI scores were associated with greater CMV seropositivity, ranging from 51.7% for the least deprived to 75.5% for the most deprived quintiles (*P* < .001), independent of age, sex, or race.

**Conclusions and Relevance:**

In this cross-sectional study, an association between SDI and CMV seropositivity was observed among SOT recipients, independent of age, sex, or race and ethnicity. To optimize posttransplant outcomes in CMV seropositive recipients, efforts targeting prevention of CMV reactivation need to be prioritized in these higher-risk populations.

## Key Points

**Question:**

Does cytomegalovirus (CMV) seropositivity among recipients of solid organ transplants differ by social deprivation, potentially contributing to disparities in transplant outcomes?

**Findings:**

In this cohort of 389 288 solid organ transplant recipients, an association between greater social deprivation and CMV seropositivity was found, independent of age, sex, and race and ethnicity.

**Meaning:**

Area-level social deprivation may play an independent role in CMV seropositivity; the impact on posttransplant CMV outcomes needs further investigation.

## Teaser

This cross-sectional study compares cytomegalovirus (CMV) seroprevalence across solid organ transplant recipients at the time of transplant, assesses the the association of recipient characteristics with CMV seropositivity, and investigates associations between area-level social deprivation and the risk of CMV seropositivity among solid organ transplant recipients.

## Introduction

Cytomegalovirus (CMV) is the most common infectious complication in solid organ transplant (SOT) recipients, occurring in approximately 50% of the general US population^1^ but in up to 62% of SOT recipients.^2^ Risk in specific organ types has not been previously reported. CMV is an important cause of morbidity and mortality following SOT,^3,4,5,6^ through direct and indirect effects that contribute to adverse outcomes, including allograft dysfunction and vasculopathy.^7,8,9,10,11^ Management of CMV infection after transplant is also associated with a significant economic burden and use of resources, placing a heavy burden on often resource-limited health care settings.^12,13^ Antiviral prophylaxis or preemptive therapy recommendations are routine across organ system transplant and can reduce clinically meaningful CMV reactivations in SOT recipients, though with added cost and toxicity.^14,15^ Consequently, the development of a CMV vaccine has been prioritized as a public health priority by the US Institute of Medicine.^5,16^

Seropositivity for CMV indicating prior infection has been previously linked to socioeconomic factors, including densely crowded living conditions, contact with young children (such as daycare), and household composition, many risk factors that are correlated with poverty and race.^17,18^ Deprivation indices can provide an aggregate measure of risk factors in residential neighborhoods, reflecting socioeconomic status, household composition, and minority status and language and can thus serve as a tool to research inequities in access to transplant and posttransplant outcomes. Greater deprivation has been noted to be associated with CMV seroprevalence in populations not undergoing transplant.^19^ Moreover, in South Africa, CMV seropositive SOT recipients experiencing deprivation have worse outcomes than those in low-risk areas.^20^ However, marked differences in disparities between South Africa and the US limit generalizability of these findings. The association between measures of area-level social deprivation with CMV seroprevalence among SOT recipients in the US is not currently known.

In this study, we aimed to (1) compare CMV seroprevalence across SOT recipients at the time of transplant, (2) assess the recipient characteristics associated with CMV seropositivity, and (3) investigate associations between area-level social deprivation and the risk of CMV seropositivity among SOT recipients. Identifying risk factors for CMV seropositivity can help in prioritizing interventions for prevention of complications associated with CMV among SOT recipients.

## Methods

The study was reviewed and approved by the Colorado Multiple Institutional Review Board, which waived the need for informed consent because of the use of deidentified registry data. This report follows the Strengthening the Reporting of Observational Studies in Epidemiology (STROBE) reporting guideline for observational studies.

### Data Source

This cross-sectional study used data from the Scientific Registry of Transplant Recipients (SRTR).^21^ The SRTR data system includes data on all donors, wait-listed candidates, and transplant recipients in the US, submitted by the members of the Organ Procurement and Transplantation Network (OPTN). The Health Resources and Services Administration of the US Department of Health and Human Services provides oversight to the activities of the OPTN and SRTR contractors. The data reported herein have been supplied by the Hennepin Healthcare Research Institute as the contractor for the SRTR.

Area-level social deprivation was calculated using the 2017 Social Deprivation Index (SDI) from the Robert Graham Center.^22^ This index is calculated using the 2013-2017 American Community Survey and is a composite of the following 7 demographic characteristics: (1) percentage of population living in poverty, (2) percentage of population aged 25 years or older with less than 12 years of education, (3) percentage of population aged 16 to 64 years who are not employed, (4) percentage of households living in renter-occupied housing units, (5) percentage of households living in crowded housing units, (6) percentage of single-parent families with dependents younger than 8 years, and (7) percentage of households with no vehicle.^22^ The SDI is scored from 0 to 100, with higher scores indicating greater social deprivation. The area-level SDI was obtained at the zip code tabulation area (ZCTA) level using SRTR recipient permanent zip code data, and a ZCTA–zip code crosswalk was used to merge with SRTR data at the zip code level.^23^ Last, we obtained the 2013 rural-urban continuum codes (RUCCs) from the US Department of Agriculture’s Economic Research Service.^24^ The RUCCs distinguish US metropolitan counties by the population size of their metropolitan area, and nonmetropolitan counties by their degree of urbanization and adjacency to a metropolitan area.

### Study Population

We identified adults (aged ≥18 years) who received a transplant between January 1, 2008, and May 31, 2022, and excluded adults who received organs from multiple donors, who had repeat transplants, and with missing CMV serostatus. For analyses of associations between CMV serostatus and area-level SDI, we only included patients with available permanent zip codes who resided within the 50 states or Washington, DC (eFigure 1 in Supplement 1 provides a population workflow diagram with the number of patients retained). Area-level SDI could not be calculated for those living in US territories, zip codes that fall within military bases, or those who received transplants other than pancreas, intestine, or kidney and pancreas (permanent zip code is not reported by the SRTR for these transplants). Additional recipient characteristics at the time of transplant were abstracted from the SRTR, including age, sex, race and ethnicity, body mass index (BMI), and, among kidney transplant recipients, years receiving dialysis.^25^

### Variable Definitions

Race and ethnicity are reported by transplant centers in standard OPTN forms including in the SRTR. For this analysis, we categorized the following racial and ethnic groups: Black, Hispanic, non-Hispanic White, and other. The other race and ethnicity group included American Indian or Alaska Native, Arab or Middle Eastern, Asian, Indian from the subcontinent, Native Hawaiian or Other Pacific Islander, and multiracial.

We categorized area-level SDI scores by quintiles (0-19, 20-39, 40-59, 60-79, 80-100). The RUCC scores range from 1 to 9 and distinguished metropolitan counties by the population size of the metropolitan area, and nonmetropolitan counties by the degree of urbanization and adjacency to a metropolitan area; we reported these as metropolitan area (codes 1-3), nonmetropolitan urban area (codes 4-7), and nonmetropolitan rural area (codes 8 and 9).

### Statistical Analysis

Data were analyzed from April 10 to October 25, 2023. Continuous measures were summarized using means and SDs and were compared between CMV seropositive and seronegative recipients using unpaired, unequal variance, 2-tailed *t* tests. Categorical factors were compared using Pearson χ^2^ tests.

We used multivariate imputation by chained equations to impute 5 datasets with complete data. The multiple imputation included the following a priori variables: age, sex, race and ethnicity, BMI, primary insurance, diabetes type, HIV status, hepatitis C virus (HCV) status, transplant year, organ type, and CMV serostatus. All models were fitted on each of the 5 imputed datasets, and parameter estimates were combined.

Generalized linear models were used to estimate prevalence ratios (PRs), with the outcome of recipient CMV serostatus using a logit link for binary outcomes (CMV seropositive and CMV seronegative). All models included the following variables: age, sex, race and ethnicity, BMI category, primary insurance, diabetes type, HIV status, HCV status, transplant year, and organ type (either adjusting for or stratified by). We tested 2-way interactions between organ type and all other variables in the model and found significant interactions (*P* < .05) with sex, BMI, insurance, and HCV status. Hence, we report an overall model as well as models stratified by organ type; stratified models included the variables listed above plus time receiving dialysis for kidney transplant recipients or receiving or not receiving dialysis for liver, heart, lung, kidney-liver combination, kidney-pancreas combination, and other multiorgan recipients.

In a subgroup analysis, among patients with available zip codes, we explored the association between area-level SDI and CMV serostatus adjusting for the variables above. We tested 2-way interactions between area-level SDI and all other variables in the model and found significant interactions (*P* < .05) with age, sex, and race and ethnicity. Hence, we report an overall model as well as models stratified by these 3 recipient characteristics.

All tests were 2-tailed and performed at a significance level of *P* < .05. No adjustments for multiple comparisons were made. Analyses were performed using SAS software, version 9.4 (SAS Institute Inc).

## Results

A total of 389 288 SOT recipients were included. Of those, 371 160 recipients (95.3%) had available zip code data and were also included in the area-level SDI analyses (eFigure 1 in Supplement 1). Overall, the mean (SD) age was 53.3 (13.0) years; 63.0% were male and 37.0% were female; and 21.4% were Black, 15.2% were Hispanic White, 56.2% were non-Hispanic White, and 7.2% were categorized as other. Most patients were kidney transplant recipients (56.9%), followed by liver (21.7%), heart (8.2%), and lung (7.3%) recipients. Data were missing for the following variables: HIV status (4.6%), area-level SDI (4.6%), education (4.2%), RUCC (4.0%), HCV status (1.8%), BMI (1.0%), diabetes type (0.3%), primary insurance (0.02%), and citizenship status (0.01%).

A total of 244 058 patients (62.7%) were CMV seropositive. Patient characteristics by CMV serostatus are detailed in the Table. Seropositivity for CMV increased with age, was higher among female vs male recipients (69.9% vs 58.5%; *P* < .001) and differed by race and ethnicity, with 74.8% CMV seropositivity among Black patients, 80.2% among Hispanic White patients, 50.4% among non-Hispanic White patients (*P* < .001) and stratified by organ type in eFigure 2 in Supplement 1). By organ type, CMV seropositive prevalence was highest among kidney and liver (66.2%), kidney (64.5%), and liver (63.6%) transplant recipients. In comparison, heart (57.4%) and lung (56.2%) transplant recipients had lower CMV seropositive prevalence. Prevalence of CMV seropositivity varied across OPTN regions, with the highest prevalence in regions 5 (71.0%), 4 (69.8%), and 3 (67.7%).

**Table.** Characteristics of Transplant Recipients by Pretransplant CMV Serostatus

Factor	CMV seronegative (n = 145 230)		CMV seropositive (n = 244 058)		*P* value
	No. missing	No. (%) of recipients^a^	No. missing	No. (%) of recipients^a^	
Age at transplant, mean (SD), y	0	52.0 (13.5)	0	54.0 (12.7)	<.001^b^
Age category, y	0		0		
18-39	NA	28 339 (43.6)	NA	36 598 (56.4)^c^	<.001^e^
40-49		26 116 (38.6)		41 465 (61.4)^c^	
50-59		41 319 (37.0)		70 254 (63.0)^d^	
60-69		40 209 (34.3)		76 886 (65.7)^c^	
≥70		9247 (32.9)		18 855 (67.1)^c^	
Sex	0		0		
Female	NA	43 298 (30.1)	NA	100 566 (69.9)	<.001^e^
Male		101 932 (41.5)		143 492 (58.5)	
Race and ethnicity	0		0		
Black	NA	20 918 (25.2)	NA	62 200 (74.8)^c^	<.001^e^
Hispanic White		11 719 (19.8)		47 584 (80.2)^c^	
Non-Hispanic White		108 617 (49.6)		110 177 (50.4)^c^	
Other^f^		3976 (14.2)		24 097 (85.8)^c^	
Educational level	5817		10 644		
High school or less	NA	53 654 (31.7)	NA	115 630 (68.3)^c^	<.001^e^
Some college		38 499 (39.6)		58 809 (60.4)^c^	
College or more		47 260 (44.5)		58 975 (55.5)^c^	
Primary insurance	28		66		
Private	NA	68 624 (43.2)	NA	90 233 (56.8)^c^	<.001^e^
Medicare		63 451 (33.9)		123 556 (66.1)^c^	
Medicaid or CHIP		9482 (29.1)		23 107 (70.9)^c^	
Other		3645 (33.9)		7096 (66.1)^c^	
BMI, mean (SD)	1356	28.2 (5.6)	2706	28.1 (5.5)	<.001^b^
Diabetes type	371		735		
None	NA	98 678 (38.5)	NA	157 826 (61.5)^c^	<.001^e^
Type I		9730 (46.9)		11 023 (53.1)^c^	
Type II		34 302 (32.8)		70 157 (67.2)^c^	
Type other or unknown		2149 (33.2)		4317 (66.8)^c^	
HIV positive	6088	354 (10.9)	11 864	2895 (89.1)	<.001^e^
HCV positive	2812	11 001 (29.6)	4184	26 218 (70.4)	<.001^e^
Receiving dialysis	1094	69 656 (33.7)	1612	137 075 (66.3)	<.001^e^
Citizenship status	16		15		
US citizen	NA	142 686 (39.1)	NA	222 211 (60.9)^c^	<.001^e^
Noncitizen US resident		2074 (10.3)		18 156 (89.7)^c^	
Noncitizen and nonresident in the US		454 (11.0)		3676 (89.0)^c^	
Transplant year	0		0		
2008-2012	NA	40 255 (36.1)	NA	71 213 (63.9)^c^	<.001^e^
2013-2017		47 751 (37.3)		80 104 (62.7)	
2018-2022		57 224 (38.2)		92 741 (61.8)^c^	
Transplant organ type	0		0		
Kidney	NA	78 652 (35.5)	NA	142 721 (64.5)^c^	<.001^e^
Liver		30 826 (36.4)		53 833 (63.6)^c^	
Heart		13 600 (42.6)		18 297 (57.4)^c^	
Lung		12 435 (43.8)		15 966 (56.2)^c^	
Pancreas		520 (55.6)		415 (44.4)^c^	
Intestine		222 (47.4)		246 (52.6)^c^	
Kidney and pancreas		5177 (47.0)		5848 (53.0)^c^	
Kidney and liver		2438 (33.8)		4773 (66.2)^c^	
Other multiorgan		1360 (41.0)		1959 (59.0)^c^	
OPTN region No.^g^	0		0		
1	NA	7885 (52.1)	NA	7239 (47.9)^c^	<.001^e^
2		20 650 (43.8)		26 479 (56.2)^c^	
3		17 322 (32.3)		36 328 (67.7)^c^	
4		11 507 (30.2)		26 594 (69.8)^c^	
5		17 891 (29.0)		43 892 (71.0)^c^	
6		4767 (37.5)		7951 (62.5)	
7		13 960 (40.8)		20 245 (59.2)^c^	
8		10 321 (42.1)		14 206 (57.9)^c^	
9		9942 (38.6)		15 813 (61.4)^c^	
10		15 863 (44.5)		19 763 (55.5)^c^	
11		15 122 (37.2)		25 548 (62.8)	
Area-level SDI, mean (SD)^h^	7666	45.2 (28.2)	10 097	55.8 (29.1)	<.001^b^
Area-level SDI, quintile	7666		10 097		
1 (least deprived)	NA	32 704 (48.3)	NA	34 993 (51.7)^c^	<.001^e^
2		31 061 (43.2)		40 839 (56.8)^c^	
3		28 057 (38.9)		44 064 (61.1)^c^	
4		24 709 (33.4)		49 181 (66.6)^c^	
5 (most deprived)		21 033 (24.5)		64 884 (75.5)^c^	
RUCC (2013)	6802		8781		
Metropolitan	NA	119 307 (36.5)	NA	207 309 (63.5)^c^	<.001^e^
Nonmetropolitan urban		17 204 (40.6)		25 191 (59.4)^c^	
Nonmetropolitan completely rural		1917 (40.8)		2777 (59.2)^c^	

Abbreviations: BMI, body mass index (calculated as the weight in kilograms divided by the height in meters squared); CMV, cytomegalovirus; HCV, hepatitis C infection; NA, not applicable; OPTN, Organ Procurement and Transplantation Network; RUCC, rural-urban continuum code; SDI, social deprivation index.

^a^ Calculated as row percentages.

^b^ Calculated using the Satterthwaite *t* test.

^c^ Significantly different than all other categories combined with *P* < .001.

^d^ Significantly different than all other categories combined with *P* < .05.

^e^ Calculated using the Pearson χ^2^ test.

^f^ Includes American Indian or Alaska Native, Arab or Middle Eastern, Asian, Indian from the subcontinent, Native Hawaiian or Other Pacific Islander, and multiracial.

^g^ Region 1 includes Connecticut, Maine, Massachusetts, New Hampshire, Rhode Island, and Eastern Vermont; region 2, Delaware, Washington, DC, Maryland, New Jersey, Pennsylvania, West Virginia, and Northern Virginia; region 3, Alabama, Arkansas, Florida, Georgia, Louisiana, Mississippi, and Puerto Rico; region 4, Oklahoma and Texas; region 5, Arizona, California, Nevada, New Mexico, and Utah; region 6, Alaska, Hawaii, Idaho, Montana, Oregon, and Washington; region 7, Illinois, Minnesota, North Dakota, South Dakota, and Wisconsin; region 8, Colorado, Iowa, Kansas, Missouri, Nebraska, and Wyoming; region 9, New York and Western Vermont; region 10, Indiana, Michigan, and Ohio; and region 11, Kentucky, North Carolina, South Carolina, Tennessee, and Virginia.

^h^ Ranges from 0 to 100, with higher SDI indicating greater social deprivation.

**Figure 1.**
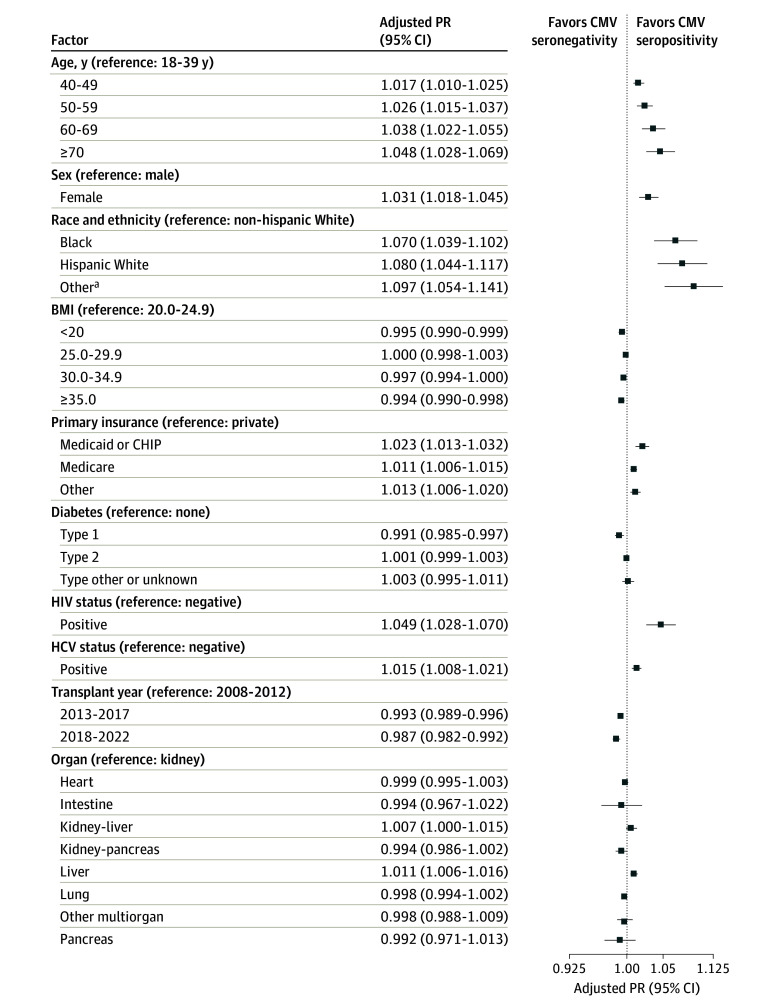
Adjusted Prevalence Ratio (PR) of Recipient Characteristics and Cytomegalovirus (CMV) Seropositivity at the Time of Transplant Squares represent the PR estimates; the error bars, 95% CIs. The x-axis is plotted in a log scale. Prevalence ratios are adjusted for age category, sex, race and ethnicity, body mass index (BMI; calculated as the weight in kilograms divided by the height in meters squared) category, primary insurance, diabetes type, HIV status, hepatitis C virus (HCV) status, transplant year, and organ type. CHIP indicates Children’s Health Insurance Program. ^a^Includes American Indian or Alaska Native, Arab or Middle Eastern, Asian, Indian from the subcontinent, Native Hawaiian or Other Pacific Islander, and multiracial.

In multivariable analysis, we found a higher independent prevalence of CMV seropositivity with increasing age, among women, and among patients with Medicaid, Medicare, or other, nonprivate insurance (Figure 1). The prevalence ratio for CMV serostatus was also higher among Black and Hispanic White patients and those of other race or ethnicity compared with non-Hispanic White patients (Figure 1). When stratified by specific organ type, the associations of CMV seropositivity with sex, race, and ethnicity were consistent across all organs and for the duration of the study period (2008-2022) (eTable 1 in Supplement 1). Other factors associated with an increased prevalence of CMV seropositivity included HIV or HCV seropositivity and liver (vs kidney) transplant.

### Associations Between Area-Level SDI and CMV

As shown in the Table, the mean (SD) area-level SDI was greater for those who were CMV seropositive (55.8 [29.1]) vs CMV seronegative (45.2 [28.2]; *P* < .001). Greater SDI was associated with greater CMV seropositivity, ranging from 51.7% for least deprived to 75.5% for most deprived quintiles (*P* < .001), independent of age, sex, or race. Patients residing in higher area-level SDI quintile areas had higher CMV seropositivity across all organ types (eFigure 2 in Supplement 1). Seropositivity for CMV was slightly higher for urban metropolitan areas (63.5%) compared with urban nonmetropolitan (59.4%) and rural (59.2%) areas (Table) and by organ type (eFigure 2 in Supplement 1).

**Figure 2.**
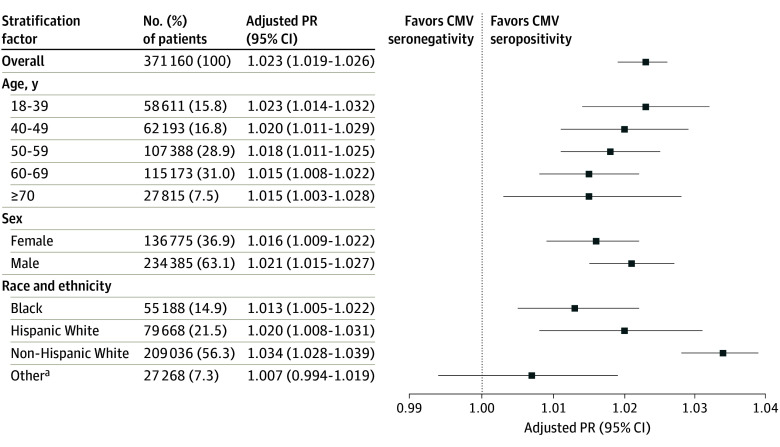
Recipient Area-Level Social Deprivation Index (SDI) and Cytomegalovirus (CMV) Seropositivity Greater SDI (quintile 5 vs 1) is associated with greater prevalence ratios (PRs) of CMV seropositivity, though differences are apparent by recipient age, sex, and race and ethnicity at the time of transplant. Interactions between area-level SDI and factor strata were statistically significant (*P* < .001). Squares represent the PR estimates; error bars, 95% CIs. The x-axis is plotted in a log scale. Prevalence ratios are adjusted for age category, sex, race and ethnicity, body mass index category, primary insurance, diabetes type, HIV status, hepatitis C virus status, transplant year, and organ type. ^a^Includes American Indian or Alaska Native, Arab or Middle Eastern, Asian, Indian from the subcontinent, Native Hawaiian or Other Pacific Islander, and multiracial.

In multivariable analysis, we found greater prevalence of CMV seropositive status with increasing area-level SDI (prevalence ratio, 1.006 [95% CI, 1.003-1.010] for quintiles 2 vs 1; 1.013 [95% CI, 1.009-1.016] for quintiles 3 vs 1; 1.018 [95% CI, 1.015-1.021] for quintiles 4 vs 1; and 1.023 [95% CI, 1.019-1.026] for quintiles 5 vs 1; all *P* < .001) (eTable 2 in Supplement 1). We also found that the association between area-level SDI and CMV seropositivity significantly differed by age, sex, and race and ethnicity. As shown in Figure 2 (showing only comparisons between area-level SDI quintiles 5 and 1), younger patients, male patients, and non-Hispanic White patients residing in areas of higher SDI tended to have a higher prevalence of seropositivity for CMV (stratified PRs for other quintiles and by organ type are detailed in eTable 2 in Supplement 1). Residing in areas of higher area-level SDI (quintiles 3 to 5) was associated with a higher prevalence of CMV seropositivity compared with area-level SDI quintile 1 across all periods (2008-2012, 2013-2017, and 2018-2022) for all organs combined; this was also true for kidney transplant recipients but not for recipients of other organ types (eTable 2 in Supplement 1). In general, the association between area-level SDI and the prevalence of CMV seropositivity was observed in the groups with the lowest CMV seroprevalence.

## Discussion

To the best of our knowledge, this cross-sectional study is the first and largest to comprehensively compare CMV seroprevalence rates among SOT recipients in North America by organ type and across area-level SDI. We found the greatest CMV seroprevalence among kidney transplant recipients, followed by liver recipients. We found greater area-level SDI among CMV seropositive individuals compared with CMV seronegative individuals, with a greater prevalence of CMV seropositivity with each increase in area-level SDI quintile. Area-level SDI and CMV seropositivity were associated among populations with lower overall CMV burden (non-Hispanic White, younger, and male recipients). Several of our findings merit further discussion.

First, our overall CMV seropositivity rate of 62.7% among SOT recipients is similar to what has been previously reported for CMV seropositivity for SOT recipients in Canada^2^ but higher than overall age-adjusted CMV seropositivity rate of 50% in the general US population (ie, not undergoing transplant).^1^ Outside North America, CMV seropositivity rates tend to be higher, ranging from 86% to 98% among SOT recipients in China,^26,27,28^ Korea,^29^ Brazil,^30^ and Hungary.^31^ Lower rates in North America may reflect differences by socioeconomic development and in the prevalence of key exposures attributed to CMV transmission such as breastfeeding frequency and duration, crowding, and child care.^26,32^ Notably, CMV seroprevalence was highest among the kidney transplant recipients (64.5%) and in kidney and liver transplant recipients (66.2%), slightly higher than previously published data from the SRTR during the study period 2013 to 2019, with a prevalence of 59.5% among kidney transplant recipients.^33^ Our higher rate of CMV seropositivity among the kidney transplant recipients could reflect the increasing age of the population of kidney transplant recipients,^34^ though notably, the overall prevalence of CMV seropositivity has decreased in recipients in more recent years.^35^ While CMV mismatch (CMV seropositive donor and CMV seronegative recipient) places SOT recipients at the highest risk of CMV infection and complications after transplant, CMV seropositive recipient status (regardless of donor) continues to place SOT recipients at moderate risk for CMV reactivation and posttransplant complications.^15^

Next, we noted racial and ethnic disparities for CMV seropositivity in SOT recipients before considering area-level SDI, with the highest rates for CMV seropositivity among SOT recipients who were Black and Hispanic compared with non-Hispanic White. These findings are similar to prior observations, with higher proportions of CMV seropositivity among Black and Hispanic vs non-Hispanic White participants in the general (nontransplant) US population.^36^ Disparities related to race in transplant outcomes have been identified, with worse patient and graft survival reported for Black kidney transplant recipients compared with White transplant recipients.^37,38,39^

Consistent with existing literature, age was an independent factor associated with CMV seropositivity. The increasing CMV seropositivity with age is likely explained by greater cumulative exposure to CMV throughout life and is reported throughout populations undergoing transplant^26,29,30,31^ and those not undergoing transplant.^40^ High levels of CMV seropositivity among older SOT recipients also place patients at high risk for CMV reactivation.^41^ Additional studies are needed to determine the extent by which CMV seropositive serostatus may contribute to transplant outcomes in these more vulnerable SOT recipients and whether prolonged CMV prophylaxis improves long-term outcomes.

Our most novel finding was the greater CMV seropositivity among SOT recipients residing in areas of highest compared with lowest deprivation. In contrast to our findings above, associations between SDI scores and CMV seropositivity were observed among those with the lowest CMV prevalence (younger, male, and non-Hispanic White recipients). These findings suggest that social deprivation may confer additional risks for CMV seropositivity beyond those previously reported in the literature. Although we were unable to explore the association of area-level SDI with CMV treatment outcomes in this cohort, a previous study^42^ found that socioeconomic disadvantage played an important role in poor outcomes for hematopoietic stem cell transplant recipients with CMV infection. Socioeconomic status may impact a host of factors, including access to affordable housing and food, area-level health statistics, poor health literacy, medical nonadherence, access to high-quality follow-up, and cultural barriers.^39^ All these factors may affect the likelihood of achieving successful outcomes after transplant.^39^ Indeed, among children receiving liver transplants, those living in neighborhoods with higher deprivation had diminished graft and patient survival.^43^ In addition to poorer outcomes, CMV infection treatment and management presents a higher economic burden in SOT recipients.^13^ Area-level SDI may be easy to calculate as a component informing CMV risk. Whether area-level SDI is associated with CMV reactivations or could inform treatment decisions for CMV prophylaxis duration are unknown but are high-priority research areas. Determining the interaction of area-level SDI and CMV seropositivity with the outcomes of organ transplant can inform mitigating interventions to optimize transplant outcomes.

### Strengths and Limitations

Strengths of our study include a large patient cohort with over a decade of data, a population undergoing organ transplant, and available CMV serostatus data. The inclusion of zip code in the SRTR database allowed us to use a novel measure of area-level SDI to evaluate CMV and socioeconomic disparity. Area-level SDI is a composite of 7 different variables, with no direct health measures but a growing body of literature associating area-level SDI with health outcomes.

Limitations include the retrospective study design with no details on primary CMV infection and/or CMV reactivations following transplant. Although some data were missing, as is the case in registry-based studies, our sample size was still large. Since the incidence of new CMV infection could not be measured in the SRTR, all estimations were made with preexisting CMV serostatus at the time of transplant. Our study is exploratory in nature, and we did not adjust for multiple comparisons. Last, area-level SDI is one of several indices available for measuring deprivation, and our results may have differed if we had chosen a different measure. In addition, area-level SDI is calculated from the zip code, which may not accurately reflect individual-level social deprivation, particularly in zip codes that cover large heterogeneous populations.

## Conclusions

In this cross-sectional study, we found that CMV seroprevalence was associated with area-level social deprivation among SOC recipients. Taken together with previous findings that both greater area-level social deprivation and CMV seropositivity were associated with poor posttransplant outcomes, our results highlight the need to determine how much of the association of area-level social deprivation with transplant outcomes may be explained by CMV infection and devise appropriate mitigating interventions.

## Notes

Supplement 1. **eFigure 1.** Patient Selection Flowchart eFigure 2.** Preexisting CMV Seropositive Status by Race and Ethnicity, Social Deprivation Index by Organ Type, and Rural-Urban Continuum Code ** **eTable 1.** Recipient Characteristics and Pretransplant CMV Seropositivity **eTable 2.** Effect of Recipient SDI on Pretransplant CMV Seropositivity, Overall and by Recipient Age, Sex, Race and Ethnicity, and Year: Subgroup Analysis of Patients With SDI Data

Supplement 2.
Data Sharing Statement
