# Supplementary material for: Area-Level Social Deprivation and Cytomegalovirus Seropositivity at the Time of Solid Organ Transplant
Source: JAMA Netw Open. 2024 Oct 7;7(10):e2437878. doi: 10.1001/jamanetworkopen.2024.37878 (PMC11581662; doi:10.1001/jamanetworkopen.2024.37878)
Supplement: Supplement 1. — eFigure 1. Patient Selection Flowchart eFigure 2. Preexisting CMV Seropositive Status by Race and Ethnicity, Social Deprivation Index by Organ Type, and Rural-Urban Continuum Code eTable 1. Recipient Characteristics and Pretransplant CMV Seropositivity eTable 2. Effect of Recipient SDI on Pretransplant CMV Seropositivity, Overall and by Recipient Age, Sex, Race and Ethnicity, and Year: Subgroup Analysis of Patients With SDI Data [file jamanetwopen-e2437878-s001.pdf]

Supplementary Online Content

Abidi MZ, Lopez R, Arrigain S, et al. Area-Level Social deprivation and cytomegalovirus seropositivity at the time of solid organ transplant. *JAMA Netw Open*. 2024;7(10):e2437878. doi:10.1001/jamanetworkopen.2024.37878

eFigure 1. Patient Selection Flowchart

eFigure 2. Preexisting CMV Seropositive Status by Race and Ethnicity, Social Deprivation Index by Organ Type, and Rural-Urban Continuum Code

eTable 1. Recipient Characteristics and Pretransplant CMV Seropositivity

eTable 2. Effect of Recipient SDI on Pretransplant CMV Seropositivity, Overall and by Recipient Age, Sex, Race and Ethnicity, and Year: Subgroup Analysis of Patients With SDI Data

This supplementary material has been provided by the authors to give readers additional information about their work.

© 2024 Abidi MZ et al. *JAMA Network Open*.

eFigure 1. Patient Selection Flowchart

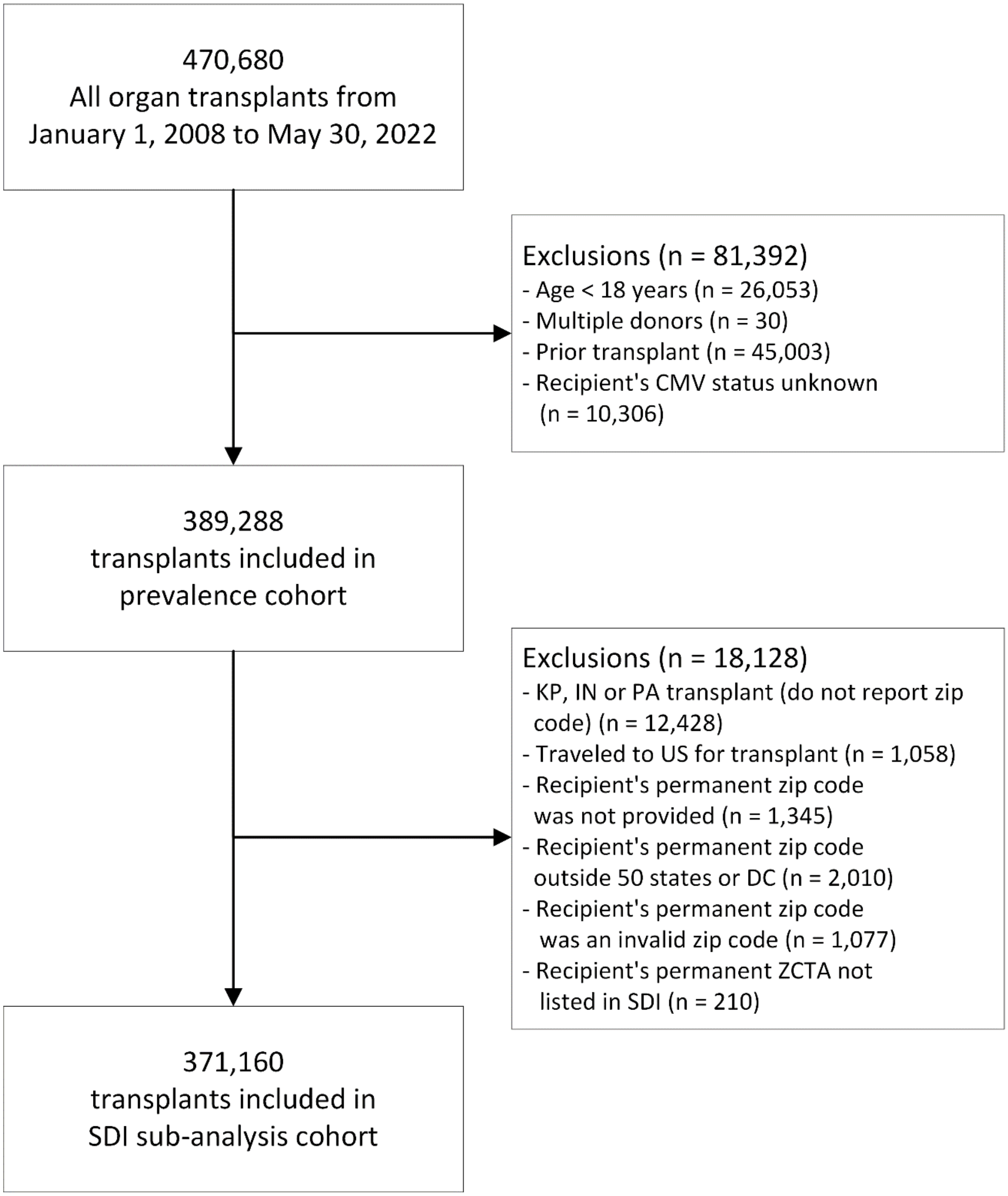

© 2024 Abidi MZ et al. *JAMA Network Open*.

eFigure 2. Preexisting CMV Seropositive Status by Race and Ethnicity, Social Deprivation Index by Organ Type, and Rural-Urban Continuum Code

CMV+ seroprevalence is described across (A) Hispanic white, non-Hispanic white, black and "others", (B) the social deprivation index quartiles ranging from 1 (least deprived), to 5 (most deprived) quartile, and (C) Urban metropolitan, Urban non-metropolitan, and rural areas, by organ type.

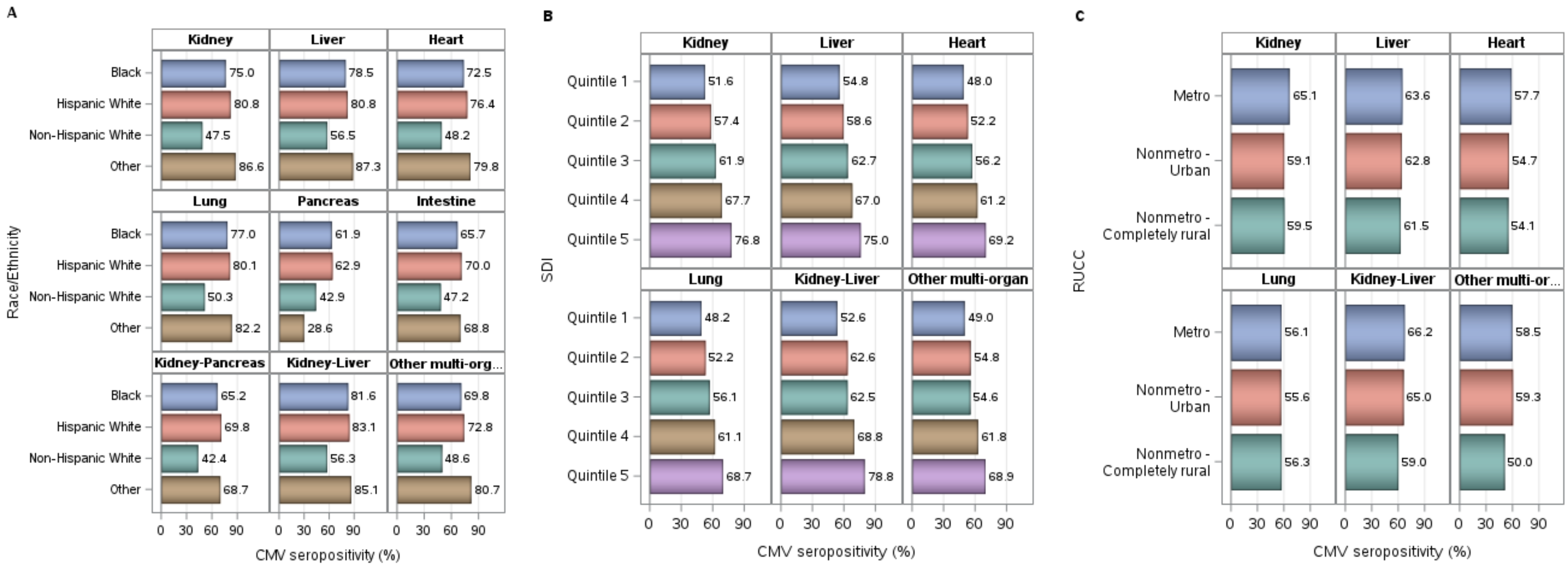

© 2024 Abidi MZ et al. *JAMA Network Open*.

eTable 1. Recipient Characteristics and Pretransplant CMV Seropositivity

A. By organ type (kidney, liver, heart)

Factor	Kidney		Liver		Heart	
	**aPR† (95% CI)**	**p-value**	**aPR† (95% CI)**	**p-value**	**aPR† (95% CI)**	**p-value**
Age (years)						
40 - 49 vs. 18 - 39	1.022 (1.011, 1.033)	***0.002***	1.011 (1.002, 1.020)	***0.016***	1.030 (1.013, 1.048)	***<0.001***
50 - 59 vs. 18 - 39	1.031 (1.015, 1.047)	***0.004***	1.020 (1.011, 1.028)	***<0.001***	1.048 (1.029, 1.068)	***<0.001***
60 - 69 vs. 18 - 39	1.048 (1.024, 1.074)	***0.005***	1.032 (1.023, 1.042)	***<0.001***	1.068 (1.045, 1.092)	***<0.001***
70+ vs. 18 - 39	1.065 (1.032, 1.100)	***0.004***	1.039 (1.025, 1.053)	***<0.001***	1.063 (1.033, 1.093)	***<0.001***
Sex						
Female vs. Male	1.036 (1.018, 1.055)	***0.005***	1.037 (1.030, 1.044)	***<0.001***	1.045 (1.030, 1.059)	***<0.001***
Race/Ethnicity						
Black vs. Non-Hispanic White	1.090 (1.043, 1.139)	***0.005***	1.059 (1.047, 1.071)	***<0.001***	1.103 (1.071, 1.137)	***<0.001***
Hispanic White vs. Non-Hispanic White	1.110 (1.052, 1.171)	***0.006***	1.062 (1.051, 1.073)	***<0.001***	1.116 (1.080, 1.154)	***<0.001***
Other‡ vs. Non-Hispanic White	1.127 (1.059, 1.199)	***0.006***	1.088 (1.071, 1.105)	***<0.001***	1.135 (1.093, 1.179)	***<0.001***
BMI						
<20 vs. 20 - 24.9	0.994 (0.987, 1.001)	0.11	0.997 (0.986, 1.009)	0.63	1.003 (0.985, 1.021)	0.76
25 - 29.9 vs. 20 - 24.9	1.000 (0.997, 1.004)	0.89	0.999 (0.993, 1.005)	0.70	1.004 (0.994, 1.014)	0.44
30 - 34.9 vs. 20 - 24.9	0.995 (0.990, 0.999)	***0.028***	0.998 (0.992, 1.005)	0.61	1.002 (0.991, 1.014)	0.71
35+ vs. 20 - 24.9	0.990 (0.983, 0.996)	***0.004***	0.999 (0.991, 1.006)	0.69	0.997 (0.981, 1.014)	0.75
Primary insurance						
Medicaid/CHIP vs. Private	1.029 (1.015, 1.043)	***0.002***	1.020 (1.013, 1.027)	***<0.001***	1.032 (1.017, 1.048)	***<0.001***
Medicare vs. Private	1.007 (1.003, 1.012)	***0.002***	1.012 (1.006, 1.017)	***<0.001***	1.018 (1.008, 1.028)	***<0.001***
Other vs. Private	1.013 (1.003, 1.024)	***0.014***	1.010 (0.999, 1.020)	0.073	1.016 (0.995, 1.037)	0.13
Diabetes						
Type I vs. No diabetes	0.989 (0.981, 0.998)	***0.014***	1.004 (0.985, 1.023)	0.68	1.002 (0.971, 1.035)	0.88
Type II vs. No diabetes	1.001 (0.997, 1.004)	0.63	0.999 (0.994, 1.005)	0.82	1.004 (0.995, 1.014)	0.37

© 2024 Abidi MZ et al. *JAMA Network Open*.

eTable 1. Recipient Characteristics and Pretransplant CMV Seropositivity

A. By organ type (kidney, liver, heart)

Factor	Kidney		Liver		Heart	
	**aPR† (95% CI)**	**p-value**	**aPR† (95% CI)**	**p-value**	**aPR† (95% CI)**	**p-value**
Type other/unknown vs. No diabetes	1.004 (0.992, 1.015)	0.55	1.007 (0.990, 1.023)	0.43	1.012 (0.977, 1.049)	0.50
HIV status						
Positive vs. Negative	1.061 (1.029, 1.095)	***0.003***	1.058 (1.029, 1.087)	***<0.001***	1.088 (1.031, 1.149)	***0.002***
HCV status						
Positive vs. Negative	1.012 (1.003, 1.021)	***0.008***	1.021 (1.015, 1.026)	***<0.001***	1.010 (0.982, 1.038)	0.49
Dialysis						
Yes vs. No	---	---	1.012 (1.005, 1.020)	***<0.001***	1.002 (0.973, 1.033)	0.88
Time on dialysis						
>0 - 11.9 months vs. No dialysis	1.007 (1.001, 1.013)	***0.014***	---	---	---	---
12 - 23.9 months vs. No dialysis	1.013 (1.006, 1.020)	***<0.001***	---	---	---	---
24 - 47.9 months vs. No dialysis	1.014 (1.006, 1.021)	***0.001***	---	---	---	---
48 - 71.9 months vs. No dialysis	1.015 (1.007, 1.023)	***0.001***	---	---	---	---
72+ months vs. No dialysis	1.019 (1.009, 1.029)	***0.001***	---	---	---	---
Transplant year						
2013 - 2017 vs. 2008 - 2012	0.992 (0.987, 0.997)	***0.002***	0.991 (0.985, 0.996)	***<0.001***	0.982 (0.972, 0.993)	***0.002***
2018 - 2022 vs. 2008 - 2012	0.985 (0.978, 0.993)	***0.002***	0.982 (0.976, 0.988)	***<0.001***	0.968 (0.956, 0.981)	***<0.001***

† Adjusted for age category, sex, race/ethnicity, BMI category, primary insurance, diabetes type, HIV status, HCV status, dialysis yes vs no (for liver, heart, lung, kidney-liver, and other multi-organ), time on dialysis (for kidney), and transplant year.
‡ The Other race/ethnicity group includes American Indian or Alaska Native, Arab or Middle Eastern, Asian, Indian sub-continent, Native Hawaiian or Other Pacific Islander, and multi-racial
CI: confidence interval; CMV: cytomegalovirus; aPR: adjusted prevalence ratio

© 2024 Abidi MZ et al. *JAMA Network Open*.

B. By organ type (lung, pancreas, intestine)

Factor	Lung		Pancreas		Intestine	
	aPR† (95% CI)	p-value	aPR† (95% CI)	p-value	aPR† (95% CI)	p-value
Age (years)						
40 - 49 vs. 18 - 39	1.048 (1.026, 1.071)	***<0.001***	1.047 (0.944, 1.162)	0.39	0.994 (0.847, 1.166)	0.94
50 - 59 vs. 18 - 39	1.071 (1.051, 1.091)	***<0.001***	1.061 (0.950, 1.186)	0.29	1.189 (1.013, 1.395)	0.034
60 - 69 vs. 18 - 39	1.079 (1.060, 1.099)	***<0.001***	1.108 (0.911, 1.349)	0.30	1.225 (0.989, 1.519)	0.063
70+ vs. 18 - 39	1.084 (1.060, 1.109)	***<0.001***	1.187 (0.470, 2.997)	0.72	0.073 (0.000, 629.264)	0.57
Sex						
Female vs. Male	1.051 (1.041, 1.061)	***<0.001***	1.141 (1.041, 1.249)	***0.005***	1.059 (0.937, 1.197)	0.36
Race/Ethnicity						
Black vs. Non-Hispanic White	1.114 (1.098, 1.131)	***<0.001***	1.141 (0.936, 1.391)	0.19	1.169 (0.977, 1.399)	0.087
Hispanic White vs. Non-Hispanic White	1.124 (1.107, 1.142)	***<0.001***	1.223 (1.000, 1.496)	0.050	1.183 (0.943, 1.483)	0.15
Other‡ vs. Non-Hispanic White	1.143 (1.119, 1.168)	***<0.001***	0.840 (0.477, 1.478)	0.55	1.152 (0.793, 1.673)	0.46
BMI						
<20 vs. 20 - 24.9	0.988 (0.973, 1.004)	0.14	0.963 (0.795, 1.166)	0.70	1.019 (0.877, 1.184)	0.81
25 - 29.9 vs. 20 - 24.9	1.002 (0.991, 1.013)	0.69	0.976 (0.883, 1.078)	0.63	1.055 (0.910, 1.224)	0.48
30 - 34.9 vs. 20 - 24.9	0.996 (0.983, 1.010)	0.59	1.020 (0.897, 1.160)	0.76	0.971 (0.699, 1.349)	0.86
35+ vs. 20 - 24.9	1.003 (0.963, 1.044)	0.88	0.869 (0.527, 1.434)	0.58	0.989 (0.567, 1.724)	0.97
Primary insurance						
Medicaid/CHIP vs. Private	1.043 (1.024, 1.061)	***<0.001***	1.046 (0.900, 1.217)	0.56	1.156 (0.968, 1.380)	0.11
Medicare vs. Private	1.019 (1.009, 1.030)	***<0.001***	1.042 (0.931, 1.166)	0.47	1.058 (0.914, 1.225)	0.45
Other vs. Private	1.037 (1.013, 1.061)	***0.002***	1.228 (0.808, 1.867)	0.34	0.993 (0.680, 1.450)	0.97
Diabetes						
Type I vs. No diabetes	0.982 (0.950, 1.015)	0.29	1.091 (0.486, 2.446)	0.83	---	---
Type II vs. No diabetes	1.001 (0.988, 1.014)	0.91	1.220 (0.516, 2.880)	0.65	---	---
Type other/unknown vs. No diabetes	0.994 (0.968, 1.020)	0.64	0.892 (0.372, 2.137)	0.80	---	---
HIV status						
Positive vs. Negative	1.023 (0.978, 1.071)	0.32	0.908 (0.551, 1.498)	0.71	---	---
HCV status						
Positive vs. Negative	1.026 (0.995, 1.058)	0.11	0.928 (0.613, 1.406)	0.73	1.035 (0.766, 1.399)	0.82

© 2024 Abidi MZ et al. *JAMA Network Open*.

B. By organ type (lung, pancreas, intestine)

Factor	Lung		Pancreas		Intestine	
	**aPR† (95% CI)**	**p-value**	**aPR† (95% CI)**	**p-value**	**aPR† (95% CI)**	**p-value**
Dialysis						
Yes vs. No	0.976 (0.902, 1.056)	0.54	0.849 (0.432, 1.669)	0.64	---	---
Transplant year						
2013 - 2017 vs. 2008 - 2012	0.987 (0.975, 0.998)	***0.022***	0.965 (0.875, 1.064)	0.47	0.908 (0.777, 1.062)	0.23
2018 - 2022 vs. 2008 - 2012	0.986 (0.974, 0.997)	***0.014***	0.998 (0.890, 1.119)	0.97	0.883 (0.748, 1.044)	0.15

† Adjusted for age category, sex, race/ethnicity, BMI category, primary insurance, diabetes type, HIV status, HCV status, dialysis yes vs no (for liver, heart, lung, kidney liver, and other multi-organ), time on dialysis (for kidney), and transplant year.

‡ The Other race/ethnicity group includes American Indian or Alaska Native, Arab or Middle Eastern, Asian, Indian sub-continent, Native Hawaiian or Other Pacific Islander, and multi-racial

CI: confidence interval; CMV: cytomegalovirus; aPR: adjusted prevalence ratio

© 2024 Abidi MZ et al. *JAMA Network Open*.

C. By organ type (kidney-pancreas, kidney-liver, other multiorgan)

Factor	Kidney-Pancreas		Kidney-Liver		Other Multi-Organ	
	aPR† (95% CI)	p-value	aPR† (95% CI)	p-value	aPR† (95% CI)	p-value
Age (years)						
40 - 49 vs. 18 - 39	1.023 (1.001, 1.045)	***0.042***	1.016 (0.979, 1.054)	0.40	1.030 (0.978, 1.084)	0.26
50 - 59 vs. 18 - 39	1.038 (1.009, 1.069)	***0.013***	1.027 (0.993, 1.061)	0.12	1.050 (1.002, 1.100)	***0.041***
60 - 69 vs. 18 - 39	1.046 (0.987, 1.109)	0.13	1.036 (1.001, 1.071)	***0.041***	1.059 (1.008, 1.113)	***0.023***
70+ vs. 18 - 39	1.240 (0.848, 1.814)	0.27	1.062 (1.011, 1.115)	***0.016***	1.060 (0.954, 1.178)	0.28
Sex						
Female vs. Male	1.082 (1.032, 1.135)	***0.007***	1.032 (1.016, 1.049)	***<0.001***	1.070 (1.034, 1.108)	***<0.001***
Race/Ethnicity						
Black vs. Non-Hispanic White	1.127 (1.047, 1.213)	***0.008***	1.073 (1.048, 1.099)	***<0.001***	1.125 (1.080, 1.171)	***<0.001***
Hispanic White vs. Non-Hispanic White	1.145 (1.054, 1.245)	***0.008***	1.076 (1.053, 1.099)	***<0.001***	1.123 (1.067, 1.181)	***<0.001***
Other‡ vs. Non-Hispanic White	1.132 (1.048, 1.224)	***0.006***	1.086 (1.051, 1.122)	***<0.001***	1.189 (1.115, 1.267)	***<0.001***
BMI						
<20 vs. 20 - 24.9	0.984 (0.949, 1.020)	0.37	0.986 (0.954, 1.020)	0.42	0.960 (0.911, 1.012)	0.13
25 - 29.9 vs. 20 - 24.9	1.003 (0.985, 1.021)	0.77	0.999 (0.980, 1.019)	0.96	1.015 (0.978, 1.053)	0.44
30 - 34.9 vs. 20 - 24.9	0.992 (0.967, 1.019)	0.58	1.003 (0.981, 1.025)	0.81	0.994 (0.950, 1.040)	0.79
35+ vs. 20 - 24.9	0.984 (0.913, 1.061)	0.67	1.006 (0.980, 1.032)	0.65	0.974 (0.907, 1.046)	0.47
Primary insurance						
Medicaid/CHIP vs. Private	1.036 (0.997, 1.076)	0.074	1.022 (0.997, 1.048)	0.087	1.043 (0.990, 1.099)	0.11
Medicare vs. Private	1.008 (0.989, 1.028)	0.40	1.013 (0.996, 1.031)	0.13	1.023 (0.989, 1.058)	0.19
Other vs. Private	1.004 (0.934, 1.080)	0.91	1.024 (0.978, 1.072)	0.31	1.087 (0.995, 1.187)	0.063
Diabetes						
Type I vs. No diabetes	0.906 (0.714, 1.150)	0.42	1.007 (0.951, 1.066)	0.82	0.988 (0.906, 1.078)	0.79
Type II vs. No diabetes	0.936 (0.738, 1.185)	0.58	0.992 (0.976, 1.009)	0.37	1.016 (0.982, 1.051)	0.37
Type other/unknown vs. No diabetes	0.903 (0.703, 1.158)	0.42	1.000 (0.946, 1.057)	0.99	0.943 (0.849, 1.046)	0.27
HIV status						
Positive vs. Negative	1.075 (1.006, 1.149)	***0.033***	1.045 (0.975, 1.120)	0.21	1.051 (0.933, 1.184)	0.41
HCV status						
Positive vs. Negative	1.046 (0.990, 1.105)	0.11	1.022 (1.003, 1.041)	***0.026***	1.022 (0.949, 1.100)	0.57

© 2024 Abidi MZ et al. *JAMA Network Open*.

C. By organ type (kidney-pancreas, kidney-liver, other multiorgan)

Factor	Kidney-Pancreas		Kidney-Liver		Other Multi-Organ	
	aPR† (95% CI)	p-value	aPR† (95% CI)	p-value	aPR† (95% CI)	p-value
Dialysis						
Yes vs. No	---	---	1.002 (0.985, 1.019)	0.82	0.995 (0.961, 1.030)	0.77
Time on dialysis						
>0 - 11.9 months vs. No dialysis	1.018 (0.989, 1.048)	0.24	---	---	---	---
12 - 23.9 months vs. No dialysis	1.013 (0.985, 1.041)	0.37	---	---	---	---
24 - 47.9 months vs. No dialysis	1.018 (0.989, 1.049)	0.23	---	---	---	---
48 - 71.9 months vs. No dialysis	1.042 (0.999, 1.087)	0.058	---	---	---	---
72+ months vs. No dialysis	1.031 (0.983, 1.081)	0.21	---	---	---	---
Transplant year						
2013 - 2017 vs. 2008 - 2012	1.006 (0.986, 1.026)	0.56	0.987 (0.967, 1.007)	0.19	0.956 (0.914, 1.000)	0.050
2018 - 2022 vs. 2008 - 2012	1.000 (0.980, 1.021)	0.99	0.981 (0.961, 1.002)	0.070	0.957 (0.918, 0.998)	***0.042***

† Adjusted for age category, sex, race/ethnicity, BMI category, primary insurance, diabetes type, HIV status, HCV status, dialysis yes vs no (for liver, heart, lung, kidney liver, and other multi-organ), time on dialysis (for kidney), and transplant year.

‡ The Other race/ethnicity group includes American Indian or Alaska Native, Arab or Middle Eastern, Asian, Indian sub-continent, Native Hawaiian or Other Pacific Islander, and multi-racial

CI: confidence interval; CMV: cytomegalovirus; aPR: adjusted prevalence ratio

© 2024 Abidi MZ et al. *JAMA Network Open*.

eTable 2. Effect of Recipient SDI on Pretransplant CMV Seropositivity, Overall and by Recipient Age, Sex, Race and Ethnicity, and Year: Subgroup Analysis of Patients With SDI Data

Organ Type	Stratification Factor	Q2 vs Q1		Q3 vs Q1		Q4 vs Q1		Q5 vs Q1	
		aPR† (95% CI)	p-value	aPR† (95% CI)	p-value	aPR† (95% CI)	p-value	aPR† (95% CI)	p-value
**All organs***	Overall	1.006 (1.003, 1.010)	***<0.001***	1.013 (1.009, 1.016)	***<0.001***	1.018 (1.015, 1.021)	***<0.001***	1.023 (1.019, 1.026)	***<0.001***
	Age, years								
	18 - 39	1.009 (0.999, 1.018)	0.087	1.008 (0.999, 1.018)	0.082	1.014 (1.005, 1.024)	***0.003***	1.023 (1.014, 1.032)	***<0.001***
	40 - 49	1.000 (0.991, 1.010)	0.96	1.005 (0.996, 1.014)	0.25	1.009 (1.000, 1.018)	***0.045***	1.020 (1.011, 1.029)	***<0.001***
	50 - 59	1.005 (0.997, 1.012)	0.2	1.007 (1.000, 1.015)	0.056	1.013 (1.005, 1.020)	***<0.001***	1.018 (1.011, 1.025)	***<0.001***
	60 - 69	1.001 (0.994, 1.008)	0.82	1.007 (1.000, 1.015)	***0.041***	1.011 (1.004, 1.018)	***0.002***	1.015 (1.008, 1.022)	***<0.001***
	70+	1.002 (0.990, 1.014)	0.74	1.009 (0.997, 1.021)	0.15	1.014 (1.001, 1.026)	***0.03***	1.015 (1.003, 1.028)	***0.019***
	Sex								
	Female	1.004 (0.997, 1.011)	0.22	1.008 (1.001, 1.015)	***0.026***	1.012 (1.005, 1.019)	***<0.001***	1.016 (1.009, 1.022)	***<0.001***
	Male	1.002 (0.996, 1.008)	0.45	1.007 (1.001, 1.013)	***0.017***	1.012 (1.006, 1.018)	***<0.001***	1.021 (1.015, 1.027)	***<0.001***
	Race/Ethnicity								
	Black	1.002 (0.992, 1.012)	0.74	1.004 (0.995, 1.014)	0.39	1.009 (1.000, 1.018)	0.062	1.013 (1.005, 1.022)	***0.002***
	Hispanic White	1.003 (0.990, 1.016)	0.61	1.009 (0.996, 1.021)	0.17	1.014 (1.002, 1.026)	***0.021***	1.020 (1.008, 1.031)	***<0.001***
	Non-Hispanic White	1.010 (1.005, 1.014)	***<0.001***	1.018 (1.013, 1.023)	***<0.001***	1.026 (1.021, 1.031)	***<0.001***	1.034 (1.028, 1.039)	***<0.001***
	Other race‡	0.999 (0.986, 1.011)	0.83	0.999 (0.987, 1.012)	0.91	1.000 (0.988, 1.013)	0.96	1.007 (0.994, 1.019)	0.3

© 2024 Abidi MZ et al. *JAMA Network Open*.

eTable 2. Effect of Recipient SDI on Pretransplant CMV Seropositivity, Overall and by Recipient Age, Sex, Race and Ethnicity, and Year: Subgroup Analysis of Patients With SDI Data

Organ Type	Stratification Factor	Q2 vs Q1		Q3 vs Q1		Q4 vs Q1		Q5 vs Q1	
		aPR† (95% CI)	p-value	aPR† (95% CI)	p-value	aPR† (95% CI)	p-value	aPR† (95% CI)	p-value
	Transplant year								
	2008-2012	1.003 (0.995, 1.010)	0.48	1.007 (0.999, 1.014)	0.081	1.009 (1.002, 1.017)	***0.014***	1.013 (1.006, 1.020)	***<0.001***
	2013-2017	1.005 (0.998, 1.012)	0.17	1.008 (1.000, 1.015)	***0.036***	1.011 (1.004, 1.019)	***0.001***	1.018 (1.011, 1.025)	***<0.001***
	2018-2022	1.002 (0.996, 1.009)	0.52	1.007 (1.001, 1.014)	***0.026***	1.014 (1.007, 1.021)	***<0.001***	1.021 (1.015, 1.028)	***<0.001***
**Kidney**	Overall	1.008 (1.003, 1.014)	***<0.001***	1.016 (1.011, 1.021)	***<0.001***	1.021 (1.016, 1.027)	***<0.001***	1.029 (1.024, 1.034)	***<0.001***
	Age, years								
	18 - 39	1.013 (1.000, 1.027)	0.053	1.013 (1.000, 1.027)	***0.045***	1.020 (1.007, 1.033)	***0.003***	1.032 (1.019, 1.044)	***<0.001***
	40 - 49	1.002 (0.989, 1.014)	0.81	1.008 (0.996, 1.021)	0.18	1.011 (0.999, 1.024)	0.063	1.025 (1.013, 1.037)	***<0.001***
	50 - 59	1.007 (0.996, 1.018)	0.24	1.012 (1.001, 1.023)	***0.031***	1.020 (1.009, 1.031)	***<0.001***	1.028 (1.017, 1.038)	***<0.001***
	60 - 69	1.002 (0.991, 1.012)	0.75	1.008 (0.998, 1.018)	0.12	1.014 (1.004, 1.024)	***0.008***	1.021 (1.011, 1.031)	***<0.001***
	70+	1.003 (0.988, 1.018)	0.74	1.011 (0.995, 1.027)	0.18	1.014 (0.998, 1.029)	0.087	1.015 (1.000, 1.031)	0.054
	Sex								
	Female	1.006 (0.997, 1.015)	0.20	1.012 (1.003, 1.021)	***0.011***	1.016 (1.007, 1.025)	***<0.001***	1.022 (1.013, 1.031)	***<0.001***
	Male	1.004 (0.996, 1.012)	0.30	1.009 (1.001, 1.017)	***0.022***	1.015 (1.007, 1.023)	***<0.001***	1.026 (1.018, 1.034)	***<0.001***
	Race/Ethnicity								

© 2024 Abidi MZ et al. *JAMA Network Open*.

eTable 2. Effect of Recipient SDI on Pretransplant CMV Seropositivity, Overall and by Recipient Age, Sex, Race and Ethnicity, and Year: Subgroup Analysis of Patients With SDI Data

Organ Type	Stratification Factor	Q2 vs Q1		Q3 vs Q1		Q4 vs Q1		Q5 vs Q1	
		aPR† (95% CI)	p-value	aPR† (95% CI)	p-value	aPR† (95% CI)	p-value	aPR† (95% CI)	p-value
	Black	1.003 (0.990, 1.015)	0.68	1.007 (0.995, 1.019)	0.25	1.011 (1.000, 1.022)	0.051	1.017 (1.007, 1.028)	***0.001***
	Hispanic White	1.004 (0.987, 1.021)	0.66	1.010 (0.994, 1.027)	0.22	1.015 (0.999, 1.031)	0.062	1.023 (1.008, 1.039)	***0.003***
	Non-Hispanic White	1.013 (1.006, 1.020)	***<0.001***	1.025 (1.018, 1.032)	***<0.001***	1.034 (1.026, 1.041)	***<0.001***	1.047 (1.038, 1.057)	***<0.001***
	Other race‡	1.001 (0.986, 1.016)	0.90	1.000 (0.986, 1.015)	0.97	1.003 (0.988, 1.018)	0.71	1.009 (0.994, 1.023)	0.23
	Transplant year								
	2008-2012	1.004 (0.993, 1.015)	0.47	1.010 (0.999, 1.020)	0.072	1.013 (1.003, 1.024)	***0.013***	1.019 (1.009, 1.029)	***<0.001***
	2013-2017	1.007 (0.997, 1.018)	0.17	1.013 (1.003, 1.023)	***0.014***	1.016 (1.006, 1.026)	***0.002***	1.026 (1.016, 1.036)	***<0.001***
	2018-2022	1.005 (0.996, 1.014)	0.31	1.010 (1.001, 1.019)	***0.029***	1.019 (1.010, 1.028)	***<0.001***	1.029 (1.020, 1.039)	***<0.001***
**Liver**	Overall	1.004 (0.997, 1.011)	0.29	1.012 (1.005, 1.019)	***0.001***	1.018 (1.010, 1.025)	***<0.001***	1.021 (1.013, 1.029)	***<0.001***
	Age, years								
	18 - 39	0.998 (0.973, 1.023)	0.87	1.008 (0.983, 1.033)	0.55	1.007 (0.982, 1.032)	0.59	1.011 (0.987, 1.036)	0.36
	40 - 49	0.992 (0.970, 1.014)	0.46	0.999 (0.977, 1.020)	0.90	1.003 (0.982, 1.025)	0.75	1.013 (0.992, 1.034)	0.22
	50 - 59	1.003 (0.986, 1.020)	0.73	1.001 (0.985, 1.018)	0.87	1.006 (0.989, 1.022)	0.49	1.011 (0.995, 1.028)	0.17
	60 - 69	0.999 (0.982, 1.016)	0.89	1.007 (0.991, 1.024)	0.39	1.011 (0.995, 1.028)	0.17	1.014 (0.998, 1.031)	0.082
	70+	0.999 (0.967, 1.032)	0.95	1.007 (0.973, 1.041)	0.70	1.023 (0.989, 1.059)	0.19	1.013 (0.977, 1.051)	0.47

© 2024 Abidi MZ et al. *JAMA Network Open*.

eTable 2. Effect of Recipient SDI on Pretransplant CMV Seropositivity, Overall and by Recipient Age, Sex, Race and Ethnicity, and Year: Subgroup Analysis of Patients With SDI Data

Organ Type	Stratification Factor	Q2 vs Q1		Q3 vs Q1		Q4 vs Q1		Q5 vs Q1	
		aPR† (95% CI)	p-value	aPR† (95% CI)	p-value	aPR† (95% CI)	p-value	aPR† (95% CI)	p-value
	Sex								
	Female	1.000 (0.983, 1.017)	0.98	1.003 (0.987, 1.020)	0.72	1.009 (0.993, 1.026)	0.28	1.008 (0.992, 1.024)	0.35
	Male	0.996 (0.980, 1.012)	0.61	1.006 (0.990, 1.021)	0.49	1.011 (0.996, 1.027)	0.16	1.018 (1.002, 1.033)	***0.025***
	Race/Ethnicity								
	Black	0.996 (0.961, 1.031)	0.80	1.000 (0.968, 1.033)	0.99	1.011 (0.979, 1.043)	0.51	1.007 (0.978, 1.037)	0.64
	Hispanic White	1.002 (0.974, 1.031)	0.90	1.010 (0.983, 1.038)	0.47	1.018 (0.991, 1.044)	0.19	1.018 (0.993, 1.044)	0.16
	Non-Hispanic White	1.005 (0.995, 1.015)	0.35	1.015 (1.005, 1.026)	***0.004***	1.025 (1.014, 1.036)	***<0.001***	1.027 (1.015, 1.040)	***<0.001***
	Other race‡	0.990 (0.961, 1.020)	0.52	0.992 (0.962, 1.023)	0.61	0.988 (0.958, 1.018)	0.43	0.999 (0.969, 1.029)	0.94
	Transplant year								
	2008-2012	0.996 (0.977, 1.014)	0.64	1.001 (0.983, 1.020)	0.90	1.004 (0.986, 1.023)	0.67	1.002 (0.984, 1.021)	0.80
	2013-2017	0.997 (0.980, 1.015)	0.77	1.001 (0.984, 1.018)	0.92	1.008 (0.991, 1.025)	0.36	1.010 (0.993, 1.027)	0.24
	2018-2022	1.000 (0.984, 1.017)	0.96	1.008 (0.992, 1.025)	0.33	1.015 (0.998, 1.032)	0.080	1.022 (1.006, 1.039)	***0.008***
**Heart**	Overall	1.006 (0.994, 1.018)	0.32	1.014 (1.002, 1.026)	***0.028***	1.021 (1.008, 1.033)	***0.001***	1.026 (1.013, 1.039)	***<0.001***
	Age, years								
	18 - 39	1.008 (0.968, 1.049)	0.71	0.998 (0.959, 1.037)	0.90	1.000 (0.963, 1.039)	0.99	1.005 (0.969, 1.042)	0.79

© 2024 Abidi MZ et al. *JAMA Network Open*.

eTable 2. Effect of Recipient SDI on Pretransplant CMV Seropositivity, Overall and by Recipient Age, Sex, Race and Ethnicity, and Year: Subgroup Analysis of Patients With SDI Data

Organ Type	Stratification Factor	Q2 vs Q1		Q3 vs Q1		Q4 vs Q1		Q5 vs Q1	
		**aPR† (95% CI)**	**p-value**	**aPR† (95% CI)**	**p-value**	**aPR† (95% CI)**	**p-value**	**aPR† (95% CI)**	**p-value**
	40 - 49	0.998 (0.959, 1.038)	0.91	0.998 (0.959, 1.039)	0.94	1.026 (0.987, 1.067)	0.20	1.030 (0.991, 1.070)	0.14
	50 - 59	1.000 (0.970, 1.031)	0.99	1.016 (0.985, 1.047)	0.31	1.021 (0.991, 1.053)	0.17	1.030 (1.000, 1.061)	0.052
	60 - 69	1.000 (0.970, 1.030)	0.99	1.021 (0.991, 1.053)	0.17	1.027 (0.998, 1.057)	0.073	1.029 (0.999, 1.059)	0.057
	70+	0.986 (0.927, 1.049)	0.66	1.013 (0.953, 1.077)	0.67	1.009 (0.941, 1.082)	0.79	1.012 (0.944, 1.085)	0.74
	Sex								
	Female	1.002 (0.970, 1.035)	0.91	1.009 (0.977, 1.041)	0.59	1.022 (0.990, 1.055)	0.18	1.026 (0.994, 1.059)	0.12
	Male	0.995 (0.969, 1.021)	0.68	1.010 (0.985, 1.036)	0.45	1.011 (0.985, 1.038)	0.40	1.016 (0.990, 1.042)	0.22
	Race/Ethnicity								
	Black	1.001 (0.963, 1.040)	0.97	1.007 (0.971, 1.044)	0.72	1.008 (0.972, 1.045)	0.67	1.017 (0.982, 1.052)	0.35
	Hispanic White	0.991 (0.931, 1.056)	0.78	1.014 (0.956, 1.076)	0.64	1.025 (0.968, 1.085)	0.39	1.030 (0.976, 1.087)	0.29
	Non-Hispanic White	1.011 (0.991, 1.031)	0.30	1.013 (0.992, 1.034)	0.24	1.028 (1.005, 1.052)	***0.016***	1.036 (1.010, 1.063)	***0.006***
	Other race‡	0.990 (0.940, 1.043)	0.71	1.003 (0.951, 1.059)	0.90	1.006 (0.954, 1.060)	0.83	1.001 (0.949, 1.057)	0.96
	Transplant year								
	2008-2012	1.001 (0.967, 1.035)	0.97	1.017 (0.983, 1.051)	0.33	1.021 (0.986, 1.057)	0.24	1.029 (0.995, 1.065)	0.092
	2013-2017	1.004 (0.974, 1.036)	0.78	1.001 (0.971, 1.032)	0.94	1.014 (0.984, 1.045)	0.36	1.015 (0.984, 1.046)	0.34

© 2024 Abidi MZ et al. *JAMA Network Open*.

eTable 2. Effect of Recipient SDI on Pretransplant CMV Seropositivity, Overall and by Recipient Age, Sex, Race and Ethnicity, and Year: Subgroup Analysis of Patients With SDI Data

		Q2 vs Q1		Q3 vs Q1		Q4 vs Q1		Q5 vs Q1	
**Organ Type**	**Stratification Factor**	**aPR† (95% CI)**	**p-value**	**aPR† (95% CI)**	**p-value**	**aPR† (95% CI)**	**p-value**	**aPR† (95% CI)**	**p-value**
	2018-2022	0.993 (0.964, 1.023)	0.64	1.013 (0.984, 1.042)	0.39	1.017 (0.987, 1.047)	0.26	1.021 (0.992, 1.051)	0.16
**Lung**	Overall	1.011 (0.996, 1.026)	0.16	1.024 (1.009, 1.040)	***0.002***	1.036 (1.020, 1.053)	***<0.001***	1.040 (1.022, 1.057)	***<0.001***
	Age, years								
	18 - 39	1.013 (0.946, 1.086)	0.70	0.972 (0.907, 1.042)	0.43	1.028 (0.962, 1.099)	0.41	1.016 (0.949, 1.089)	0.64
	40 - 49	0.993 (0.930, 1.060)	0.83	1.002 (0.942, 1.067)	0.94	1.005 (0.942, 1.072)	0.88	1.040 (0.979, 1.105)	0.20
	50 - 59	1.011 (0.970, 1.054)	0.60	1.019 (0.978, 1.062)	0.36	1.034 (0.993, 1.076)	0.10	1.038 (0.997, 1.080)	0.069
	60 - 69	0.992 (0.958, 1.026)	0.63	1.009 (0.976, 1.042)	0.60	1.009 (0.976, 1.044)	0.58	1.023 (0.989, 1.058)	0.18
	70+	1.004 (0.952, 1.059)	0.89	1.010 (0.951, 1.072)	0.75	1.017 (0.960, 1.077)	0.57	1.031 (0.977, 1.089)	0.27
	Sex								
	Female	1.001 (0.967, 1.036)	0.95	1.004 (0.971, 1.039)	0.80	1.016 (0.982, 1.050)	0.36	1.017 (0.984, 1.051)	0.31
	Male	1.004 (0.968, 1.042)	0.83	1.000 (0.964, 1.038)	0.99	1.022 (0.985, 1.059)	0.25	1.043 (1.005, 1.081)	***0.026***
	Race/Ethnicity								
	Black	0.985 (0.923, 1.051)	0.65	0.967 (0.908, 1.031)	0.30	0.985 (0.927, 1.046)	0.62	0.996 (0.942, 1.053)	0.89
	Hispanic White	1.012 (0.946, 1.083)	0.73	1.009 (0.944, 1.079)	0.80	1.030 (0.967, 1.097)	0.36	1.046 (0.985, 1.110)	0.14
	Non-Hispanic White	1.021 (0.996, 1.046)	0.098	1.036 (1.011, 1.063)	***0.005***	1.064 (1.036, 1.092)	***<0.001***	1.064 (1.033, 1.097)	***<0.001***

© 2024 Abidi MZ et al. *JAMA Network Open*.

eTable 2. Effect of Recipient SDI on Pretransplant CMV Seropositivity, Overall and by Recipient Age, Sex, Race and Ethnicity, and Year: Subgroup Analysis of Patients With SDI Data

Organ Type	Stratification Factor	Q2 vs Q1		Q3 vs Q1		Q4 vs Q1		Q5 vs Q1	
		aPR† (95% CI)	p-value	aPR† (95% CI)	p-value	aPR† (95% CI)	p-value	aPR† (95% CI)	p-value
	Other race‡	0.992 (0.930, 1.059)	0.82	0.998 (0.941, 1.059)	0.94	0.998 (0.936, 1.065)	0.96	1.014 (0.947, 1.086)	0.68
	Transplant year								
	2008-2012	1.014 (0.972, 1.058)	0.52	1.019 (0.977, 1.062)	0.38	1.032 (0.989, 1.077)	0.15	1.035 (0.992, 1.079)	0.12
	2013-2017	1.018 (0.977, 1.061)	0.40	1.008 (0.967, 1.051)	0.71	1.012 (0.971, 1.056)	0.57	1.041 (0.998, 1.086)	0.062
	2018-2022	0.982 (0.945, 1.022)	0.38	0.987 (0.950, 1.026)	0.51	1.016 (0.980, 1.054)	0.39	1.024 (0.987, 1.063)	0.21
**Kidney-Liver**	Overall	1.026 (0.999, 1.054)	0.059	1.019 (0.992, 1.046)	0.18	1.033 (1.006, 1.061)	***0.018***	1.035 (1.007, 1.065)	***0.016***
	Age, years								
	18 - 39	1.028 (0.919, 1.150)	0.62	1.034 (0.926, 1.155)	0.55	1.052 (0.943, 1.175)	0.36	1.031 (0.925, 1.149)	0.58
	40 - 49	1.055 (0.969, 1.147)	0.22	1.004 (0.923, 1.091)	0.93	1.018 (0.936, 1.106)	0.68	1.044 (0.967, 1.127)	0.27
	50 - 59	1.014 (0.953, 1.080)	0.65	0.995 (0.935, 1.059)	0.88	1.012 (0.953, 1.075)	0.69	1.021 (0.963, 1.082)	0.49
	60 - 69	1.011 (0.956, 1.070)	0.69	1.010 (0.956, 1.067)	0.73	1.036 (0.983, 1.092)	0.19	1.027 (0.975, 1.083)	0.32
	70+	1.009 (0.894, 1.140)	0.88	0.990 (0.878, 1.115)	0.86	0.993 (0.867, 1.137)	0.92	0.987 (0.874, 1.115)	0.84
	Sex								
	Female	1.026 (0.963, 1.093)	0.42	1.011 (0.950, 1.076)	0.73	1.017 (0.955, 1.083)	0.60	1.020 (0.962, 1.081)	0.51
	Male	1.021 (0.963, 1.083)	0.49	1.001 (0.946, 1.060)	0.96	1.027 (0.969, 1.089)	0.37	1.024 (0.969, 1.082)	0.40

© 2024 Abidi MZ et al. *JAMA Network Open*.

eTable 2. Effect of Recipient SDI on Pretransplant CMV Seropositivity, Overall and by Recipient Age, Sex, Race and Ethnicity, and Year: Subgroup Analysis of Patients With SDI Data

Organ Type	Stratification Factor	Q2 vs Q1		Q3 vs Q1		Q4 vs Q1		Q5 vs Q1	
		aPR† (95% CI)	p-value	aPR† (95% CI)	p-value	aPR† (95% CI)	p-value	aPR† (95% CI)	p-value
	Race/Ethnicity								
	Black	1.047 (0.944, 1.162)	0.38	1.022 (0.924, 1.130)	0.67	1.041 (0.945, 1.146)	0.42	1.029 (0.942, 1.123)	0.53
	Hispanic White	0.997 (0.890, 1.118)	0.96	0.975 (0.875, 1.087)	0.65	0.990 (0.889, 1.102)	0.85	0.996 (0.900, 1.101)	0.93
	Non-Hispanic White	1.034 (0.995, 1.075)	0.091	1.029 (0.990, 1.071)	0.15	1.032 (0.991, 1.075)	0.13	1.040 (0.994, 1.087)	0.086
	Other race‡	1.016 (0.904, 1.141)	0.79	0.999 (0.891, 1.120)	0.99	1.026 (0.915, 1.151)	0.66	1.024 (0.917, 1.143)	0.68
	Transplant year								
	2008-2012	1.039 (0.961, 1.123)	0.34	0.982 (0.908, 1.061)	0.64	1.007 (0.935, 1.084)	0.86	0.996 (0.927, 1.071)	0.92
	2013-2017	1.025 (0.957, 1.097)	0.48	1.020 (0.955, 1.090)	0.56	1.031 (0.963, 1.104)	0.38	1.028 (0.965, 1.094)	0.40
	2018-2022	1.024 (0.959, 1.094)	0.48	1.010 (0.947, 1.076)	0.77	1.029 (0.963, 1.100)	0.40	1.035 (0.973, 1.101)	0.27
**Other multi-organ**	Overall	1.012 (0.955, 1.073)	0.69	0.997 (0.940, 1.056)	0.91	1.023 (0.965, 1.083)	0.44	1.019 (0.962, 1.080)	0.51
	Age, years								
	18 - 39	0.974 (0.818, 1.160)	0.77	0.949 (0.809, 1.113)	0.52	0.967 (0.831, 1.125)	0.66	0.927 (0.796, 1.080)	0.33
	40 - 49	1.000 (0.848, 1.178)	0.99	1.030 (0.884, 1.200)	0.70	0.998 (0.855, 1.166)	0.98	0.990 (0.849, 1.154)	0.90
	50 - 59	1.045 (0.921, 1.184)	0.50	0.994 (0.877, 1.127)	0.93	1.039 (0.922, 1.171)	0.53	1.029 (0.921, 1.150)	0.61
	60 - 69	1.027 (0.904, 1.168)	0.68	1.031 (0.914, 1.164)	0.62	1.034 (0.904, 1.182)	0.63	1.018 (0.901, 1.150)	0.78

© 2024 Abidi MZ et al. *JAMA Network Open*.

eTable 2. Effect of Recipient SDI on Pretransplant CMV Seropositivity, Overall and by Recipient Age, Sex, Race and Ethnicity, and Year: Subgroup Analysis of Patients With SDI Data

Organ Type	Stratification Factor	Q2 vs Q1		Q3 vs Q1		Q4 vs Q1		Q5 vs Q1	
		aPRł (95% CI)	p-value	aPRł (95% CI)	p-value	aPRł (95% CI)	p-value	aPRł (95% CI)	p-value
	70+	1.188 (0.862, 1.637)	0.29	1.033 (0.786, 1.357)	0.81	0.921 (0.658, 1.290)	0.63	1.022 (0.770, 1.357)	0.88
	Sex								
	Female	1.049 (0.893, 1.232)	0.56	1.020 (0.888, 1.172)	0.78	1.012 (0.870, 1.176)	0.88	0.984 (0.852, 1.136)	0.83
	Male	1.039 (0.933, 1.158)	0.48	0.994 (0.898, 1.099)	0.90	0.971 (0.873, 1.079)	0.58	1.009 (0.917, 1.111)	0.85
	Race/Ethnicity								
	Black	1.011 (0.874, 1.168)	0.89	0.943 (0.823, 1.082)	0.40	0.972 (0.844, 1.119)	0.69	0.970 (0.857, 1.098)	0.63
	Hispanic White	1.080 (0.860, 1.356)	0.51	1.079 (0.857, 1.357)	0.52	1.023 (0.832, 1.259)	0.83	1.028 (0.842, 1.255)	0.79
	Non-Hispanic White	1.027 (0.931, 1.133)	0.60	1.016 (0.927, 1.113)	0.74	1.019 (0.922, 1.126)	0.71	1.039 (0.933, 1.157)	0.49
	Other race‡	1.060 (0.827, 1.360)	0.64	0.995 (0.804, 1.230)	0.96	0.951 (0.740, 1.223)	0.69	0.952 (0.756, 1.200)	0.68
	Transplant year								
	2008-2012	1.043 (0.876, 1.242)	0.64	1.004 (0.853, 1.181)	0.96	1.016 (0.864, 1.196)	0.84	1.009 (0.870, 1.170)	0.91
	2013-2017	1.056 (0.920, 1.211)	0.44	1.030 (0.905, 1.172)	0.65	1.016 (0.884, 1.168)	0.82	1.033 (0.910, 1.171)	0.62
	2018-2022	1.037 (0.914, 1.177)	0.57	0.995 (0.889, 1.112)	0.93	0.981 (0.870, 1.107)	0.76	0.978 (0.871, 1.099)	0.71

*Includes kidney, liver, heart, lung, kidney-liver, and other multi-organ. Does not include pancreas, intestine, or kidney-pancreas.

ł Adjusted for age category, sex, race/ethnicity, BMI category, primary insurance, diabetes type, HIV status, HCV status, dialysis yes vs no (for liver, heart, lung, kidney liver, and other multi-organ), time on dialysis (for kidney), transplant year, recipient's residential OPTN region, and organ type (for all organs model)

‡ The Other race/ethnicity group includes American Indian or Alaska Native, Arab or Middle Eastern, Asian, Indian sub-continent, Native Hawaiian or Other Pacific Islander, and multi-racial

© 2024 Abidi MZ et al. *JAMA Network Open*.

eTable 2. Effect of Recipient SDI on Pretransplant CMV Seropositivity, Overall and by Recipient Age, Sex, Race and Ethnicity, and Year: Subgroup Analysis of Patients With SDI Data

		Q2 vs Q1		Q3 vs Q1		Q4 vs Q1		Q5 vs Q1	
**Organ Type**	**Stratification Factor**	**aPR† (95% CI)**	**p-value**	**aPR† (95% CI)**	**p-value**	**aPR† (95% CI)**	**p-value**	**aPR† (95% CI)**	**p-value**

Significant interaction between SDI and 1) age category, 2) sex, and 3) race/ethnicity (p<0.001 for all 3).

CI: confidence interval; CMV: cytomegalovirus; aPR: adjusted prevalence ratio; SDI: social deprivation index; Q: quintile

SDI quintile 1 is the least deprived and quintile 5 is the most deprived

© 2024 Abidi MZ et al. *JAMA Network Open*.
