# Supplementary material for: Area-Level Social Deprivation and Cytomegalovirus Seropositivity at the Time of Solid Organ Transplant
Source: JAMA Netw Open. 2024 Oct 7;7(10):e2437878. doi: 10.1001/jamanetworkopen.2024.37878 (PMC11581662; doi:10.1001/jamanetworkopen.2024.37878)
Supplement: Supplement 2. — Data Sharing Statement [file jamanetwopen-e2437878-s002.pdf]

Data Sharing Statement

Abidi. Area-Level Social Deprivation and Cytomegalovirus Seropositivity at the Time of Solid Organ Transplant. *JAMA Netw Open*. Published October 07, 2024.
doi:10.1001/jamanetworkopen.2024.37878

Data
Data available: No

Additional Information
Explanation for why data not available: This is SRTR data. Request will need to be verified with SRTR before data can be shared
